# Metabolic host-microbe crosstalk in stabilization of epithelial HIF

**DOI:** 10.1186/s40168-026-02431-8

**Published:** 2026-05-30

**Authors:** Alexander S. Dowdell, Rebecca L. Roer, Geetha Bhagavatula, Ian M. Cartwright, Rachel H. Cohen, Jacob A. Countess, Samuel D. Koch, J. Scott Lee, Lin Liu, Calen A. Steiner, Noah T. Thompson, Zachary F. Villamaria, Nichole Welch, Corey S. Worledge, Liheng Zhou, Andrés Vazquez-Torres, Sean P. Colgan

**Affiliations:** 1 Mucosal Inflammation Program and Division of Gastroenterology and Hepatology, Department of Medicine, University of Colorado School of Medicine, Aurora, CO USA; 2 VA Eastern Colorado Health Care System, U.S. Department of Veterans Affairs, Aurora, CO USA; 3 Department of Pediatrics, Children’s Hospital Colorado, University of Colorado, Aurora, CO USA; 4 Department of Immunology & Microbiology, University of Colorado School of Medicine, Aurora, CO USA

**Keywords:** Inflammatory bowel disease, *Escherichia coli*, Intestinal epithelium, Hypoxia-inducible factor, Host-microbe interactions, Oxygen respiration, Facultative anaerobes

## Abstract

**Background:**

The gastrointestinal tract is home to trillions of microorganisms that interact with their host in profound ways. One mechanism by which these microbes interact with their eukaryotic host is through the establishment of “physiologic hypoxia” in the intestinal mucosa, which has been shown to promote gut barrier function and homeostasis in a hypoxia-inducible factor (HIF)-dependent manner. The association between HIF and intestinal homeostasis has been long understood, as activation of HIF signaling has been shown to promote barrier function both in vitro and in vivo. Although it has been previously established that pathogenic bacteria regulate HIF stabilization and activity in the intestinal epithelium independent of short-chain fatty acid metabolism, it is not clear whether this activity extends to noninfectious and/or commensal bacterial species.

**Results:**

Here, we demonstrate that nonpathogenic, commensal strains of *Escherichia coli* stabilize HIF in intestinal epithelial cells in vitro. Further, we show that HIF is transcriptionally active in epithelia and drives a “pro-barrier” transcriptional program. This property was found to be dependent on bacterial aerobic respiration, as genetic elimination of *E. coli* aerobic respiration abolished HIF stabilization and the subsequent transcriptional phenotype. Finally, we observed stabilization of tissue HIF-1α in vivo using antibiotic-treated mice colonized with wild-type, but not respiration-deficient, *E. coli*.

**Conclusions:**

These findings demonstrate a novel ability for commensal *E. coli* to regulate intestinal homeostasis through activation of HIF and suggest that this mechanism might be a major component of the interaction between facultative anaerobes and the intestinal epithelium in the gut. In addition, we hypothesize that consumption of oxygen by enteric bacteria might be leveraged as a novel therapeutic to combat intestinal inflammation, such as that observed during inflammatory bowel disease (IBD).

Video Abstract

**Supplementary Information:**

The online version contains supplementary material available at 10.1186/s40168-026-02431-8.

## Background

Inflammatory bowel disease (IBD) is a family of conditions characterized by chronic, relapsing inflammation of the gastrointestinal tract [1]. IBD can be divided into two general sub-types (Crohn’s disease and ulcerative colitis) that share some similarities but diverge in their specific pathology and genetic risk factors [2]. The pathogenesis of IBD is a complex interplay of genetic, environmental and microbial factors; as a result, a full understanding of the pathophysiological mechanisms that drive disease in the various IBD sub-types has not yet been achieved [3]. Currently, no cure for IBD exists and existing treatments are plagued by complications such as lack of response/loss of efficacy, immunosuppression, and risk of infection/cancer [4]. As a result, there is a need for better understanding of healthy gut physiology as well as the pathological mechanisms of IBD to inform the development of novel treatments.

One striking feature of IBD is the impairment of intestinal “barrier function”, that is, the gut’s ability to selectively absorb nutrients and water while excluding microbes and toxins [5]. This loss in selective permeability has the potential to permit the translocation of microbes and microbially derived molecules (such as lipopolysaccharide), resulting in systemic inflammation [6]. Interestingly, increased gut permeability has been found to accurately predict future Crohn’s disease in asymptomatic, first-degree relatives of Crohn’s disease patients [7]. Similarly, changes in gut barrier functions have been found to be a better predictor of disease prognosis than either histologic or endoscopic remission for both ulcerative colitis and Crohn’s disease [8]. These findings underscore the importance of normal barrier function in intestinal homeostasis and suggest that potentiation of gut barrier function may be a viable strategy for the treatment of IBD.

Intestinal barrier function is regulated by numerous molecular and environmental factors, including dietary, medicinal, and genetic influences [9]. One such cell-intrinsic component to intestinal barrier function is the transcription factor family *hypoxia-inducible factor* (HIF) [10]. The HIF transcriptional unit is composed of an oxygen-labile α-subunit, of which three paralogs have been identified (HIF-1α/−2α/−3α), and an oxygen-insensitive β-subunit (HIF-1β/ARNT) [11]. The stability of HIF-α proteins is regulated post-translationally by a family of prolyl hydroxylases (PHDs) which, under conditions of replete oxygen, ferrous iron, ascorbate, and α-ketoglutarate, hydroxylates HIF-α at specific proline residues as a marker for poly-ubiquitination and proteasomal degradation [12]. Under conditions in which any of these substrates becomes limiting (most notably, during hypoxia), the hydroxylation activity of PHDs becomes compromised and, as a result, HIF-α subunits accumulate in the cytoplasm. From there, these subunits dimerize with HIF-1β/ARNT, translocate to the nucleus, and exert specific transcriptional effects that can vary by cell and tissue [11]. HIF signaling is active at homeostasis in the intestinal epithelium due to the tissue’s “physiologic hypoxia”, a state driven by a combination of factors including “counter-current” gas exchange between neighboring venous and arterial capillaries in intestinal villi and epithelial β-oxidation of bacterially derived short-chain fatty acids (SCFAs) [13]. Intestinal HIF expression drives a number of “pro-barrier” factors including intestinal trefoil factor (ITF/TFF3), claudin-1, and mucin-3A [10]. Mice deficient in intestinal HIF-1α expression, correspondingly, show increased metrics of disease in mouse models of IBD, while mice treated with compounds that stabilize HIF through inhibition of PHDs are conversely protected in such models [14–16]. These findings demonstrate that HIF signaling, through multiple mechanisms, promotes gut homeostasis and is protective during intestinal inflammation.

HIF signaling has been shown to be modulated by cell-extrinsic factors, in addition to the intrinsic regulation by PHDs. Previously, our group and others demonstrated that pathogenic bacteria stabilize HIF-1α in epithelial cells and activate HIF-1α-dependent transcriptional pathways [17]. This phenomenon was found to be true for other, diverse pathogenic microbes, including *Staphylococcus aureus*, *Yersinia enterocolitica*, *Borrelia burgdorferi*, and *Acinetobacter baumannii* [17, 18]. Our group subsequently demonstrated that HIF stabilization induced by the model invasive bacterium *Salmonella enterica* subsp. *enterica* serovar Typhimurium (hereafter, “*Salmonella* Typhimurium” or STm) activated anti-bacterial autophagy (xenophagy) in intestinal epithelial cells (IECs) in a HIF-dependent manner [19]. These findings provided a link between HIF stabilization, autophagy, and gut homeostasis; however, as our results indicated an oxygen-dependent mechanism for HIF-1α stabilization, we speculated that bacterial invasion might be dispensable for HIF-1α stabilization in IECs. In this work, we show that noninvasive *Salmonella* Typhimurium stabilizes HIF-1α to the same extent as wild-type bacteria. Further, we demonstrate that commensal *Escherichia coli* strains stabilize HIF-1α and activate HIF transcriptional programs in epithelial cells. Finally, we recapitulate our in vitro findings using antibiotic-treated mice, demonstrating that wild-type, but not respiration-deficient, *E. coli* rescues tissue HIF-1α expression in the colon. Taken together, these findings demonstrate a novel role for commensal bacteria such as *E. coli* in the regulation of intestinal homeostasis through activation of HIF signaling pathways.

## Methods

### Cell lines and bacterial cultures

HeLa cervical adenocarcinoma cells (ATCC #CCL-2) and Caco-2 C2BBe1 colorectal adenocarcinoma cells (ATCC #CRL-2102) were grown as described previously [19–21]. Briefly, cells were grown in Iscove’s Modification of DMEM (IMDM, Corning #10–016-CV) supplemented with 10% (v/v) heat-inactivated bovine calf serum (BCS, Cytiva #SH30072.03) and 1 × GlutaMAX (Thermo #35050–061) without antibiotics. hIEC-6 cells (ATCC #CRL-3266) were grown in Opti-MEM (Thermo #31985–070) supplemented with 20 mM HEPES (Thermo #15630–080), 10 mM GlutaMAX, 10 ng/mL epidermal growth factor (Corning #354052), and 4% (v/v) fetal bovine serum (ATCC #30–2021), similarly without antibiotics. All cells were grown at 37 °C, 5% CO_2_ in a humidified incubator. For experiments involving bacterial-cell co-cultures, cells were plated in 6-well tissue-culture-treated plates at approximately 2.4 × 10^5^ cells/well the day before the experiment. C2BBe1 cells were plated onto collagen-coated 6-well plates (Corning #354,400) to improve adherence. Cells were regularly tested for mycoplasma contamination using established protocols [22].

Bacterial strains used in these experiments are listed in Table 1. All bacterial strains were routinely cultured in LB-Miller broth (BD #244620) at 37 °C, 250 RPM and maintained on LB-Miller plates solidified with 1.5% (w/v) agar (BD #214010) except as indicated. Plates were freshly struck on a weekly or a biweekly basis from 15% (v/v) glycerol stocks kept at −80 °C. Antibiotics were included at the following working concentrations, as needed: 100 μg/mL carbenicillin (Sigma-Aldrich #C1389), 100 μg/mL streptomycin (Sigma-Aldrich #S6501), 100 μg/mL spectinomycin (Sigma-Aldrich #S0692), 100 μg/mL kanamycin (Sigma-Aldrich #60615), and 25 μg/mL chloramphenicol (Zymo Research #A1002-5). Experiments involving the *E. coli* Nissle strain lacking all three cytochrome oxidases and *ygiN* (*E. coli* Nissle “4KO”) were performed using BHI broth (BD #237500) and BHI agar (BD #211065). When experiments were performed that included *E. coli* Nissle 4KO, all other bacterial strains were similarly grown in BHI as a control. For cell treatments, bacterial overnight cultures were grown in 2 mL of medium with antibiotics, as necessary, using 14 mL loosely capped, round-bottom tubes. One milliliter of overnight culture was then pelleted at 8,000 × *g* for 3 min at room temperature, with the resulting supernatant removed by pipetting. The bacterial pellet was resuspended in 1 mL of sterile PBS, pH 7.4 and used immediately for cell treatments. *Lactococcus lactis* str. MG1363 was obtained from Boca Scientific (Boca Raton, FL, USA) and routinely cultured in M17 medium (BD #218561) supplemented with 0.5% (w/v) glucose (“GM17 medium”). When necessary, GM17 was solidified with 1.5% (w/v) agar to prepare plates. *L. lactis* was cultured without aeration at 30 °C and struck fresh on a weekly basis from freezer stocks.

**Table 1:** Bacterial strains used in this study

Strain	Reference	Antibiotic resistance
NEB 5-alpha	NEB #C2987	n/a
*E. coli* Nissle (wild-type)	Lab stock	n/a
*E. coli* Nissle Δ*cyoABCDE* Δ*appCB* Δ*ygiN*::*amp* (“3KO”)	This work	Ampicillin/carbenicillin
*E. coli* Nissle Δ*cyoABCDE* Δ*appCB* Δ*ygiN*::*amp* Δ*cydAB* (“4KO”)	This work	Ampicillin/carbenicillin
*E. coli* GDAR2-2	Gift from Diehl Lab [23]	Ampicillin/carbenicillin, vancomycin, metronidazole, neomycin
*Salmonella* Typhimurium SL1344 (wild-type)	Lab stock	Streptomycin
*Salmonella* Typhimurium SL1344 Δ*invA*	Gift from Vázquez-Torres Lab (University of Colorado Anschutz Medical Campus; Aurora, CO, USA) (this work)	Streptomycin, chloramphenicol
*Lactococcus lactis* str. MG1363	Boca Scientific Inc.	n/a

### Construction of mutant strains

The *invA*::*Cm* mutation was moved from *S*. Typhimurium strain 14028 s into strain SL1344 by P22-mediated transduction [24]. The resulting transductants were selected in LB agar plates containing 100 μg/ml streptomycin and 20 μg/ml chloramphenicol. The pseudolysogens were eliminated by streaking on Evans blue uranine agar plates. Genetically modified *E. coli* Nissle strains were constructed using CRISPR/Cas9 + λ-Red recombineering as described previously [25]. Strain creation was designed/planned in silico using SnapGene prior to in vitro experiments. Primer and guide RNA (gRNA) sequences used can be found in Table 2, with the latter given as the oligos needed for cloning into linearized pEcgRNA (see below). In brief, gRNA sequences were designed against the regions to be deleted (*cyoABCDE*, *appCB*, and *cydAB*) and validated against the *E. coli* Nissle genome using CHOPCHOP [26–28]. Three gRNA sequences (A, B, C) were designed and tested separately for each genomic locus to be edited. The plasmids pEcCas (Addgene #73227) and pEcgRNA (Addgene #166581) were gifts from Sheng Yang. Primers were designed to amplify the genomic regions immediately 5’ and 3’ of the regions to be deleted, with the 5’ reverse primer ending at the start codon of the first gene in the operon and the 3’ forward primer beginning at the stop codon of the last gene. These primers also contained overlaps to facilitate covalent linkage of these fragments for the creation of recombineering donor DNA. The result is that the donor DNA for λ-Red recombineering contains an in-frame deletion of the coding DNA sequence of each of the deleted genes, minimizing the likelihood of polar effects on nearby genes. PCR fragments from the 5’ and 3’ regions were amplified using high-fidelity polymerase (Q5 Hot Start, NEB) and gel-purified. Fragments were then covalently joined using NEBuilder HiFi Master Mix to reduce the chance of annealing errors; the resulting covalently linked fragment was then PCR amplified and gel-purified. pEcgRNA was linearized by digestion with BsaI-HFv2 (NEB), and the correctly sized fragment was gel-purified. Linearized pEcgRNA and gRNA oligos were ligated as described elsewhere and cloned into chemically competent NEB 5-alpha cells (NEB). Plasmids were purified from the resulting transformants and sequenced using Nanopore whole-plasmid sequencing (Quintara Biosciences). *E. coli* Nissle was made electrocompetent as described elsewhere using ice-cold 10% glycerol and stored as 30 μL aliquots at −80 °C [29]. Electrocompetent *E. coli* Nissle was first transformed with pEcCas, then subsequent electrocompetent cells were prepared with the inclusion of 10 mM L-arabinose in sub-cultures in order to induce expression of λ-Red genes. Approximately 30 ng of pEcgRNA containing the appropriate gRNA was electroporated alongside about 120 ng of full-length donor DNA to delete each operon of interest. Electroporations were performed using a Gene Pulser Xcell Electroporation System with 0.2 cm cuvettes, using the pre-programmed exponential decay setting “Bacteria—> E. coli—> 2.5 kV, 0.2 cm”. Putative knockouts were screened by PCR, with the absence of unintended mutations confirmed by Sanger sequencing of the manipulated loci (ACGT, Inc.). After confirmation of the success of each mutation, the pEcgRNA plasmid was cured using 10 mM L-rhamnose induction to allow for subsequent mutations to be made. Deletion of *ygiN* occurred as for the others except that an ampicillin resistance cassette was included as a third fragment in between the 5’ and 3’ fragments, with the stop codon of the *ampR* gene occurring in-frame and eight codons prior to the *ygiN* stop codon. The ampicillin resistance cassette was amplified from pM1s3AsG (Addgene #137921, a gift from Neel Joshi) using the given primers and covalently joined using NEBuilder HiFi Master Mix as for the other donor DNA fragments [30]. As the presence of the ampicillin resistance cassette provided a suitable means of counter-selection for wild-type *ygiN* cells, no gRNA was utilized for this knockout. Mutations were made in the order Δ*cyoABCDE*—> Δ*appCB*—> Δ*ygiN*::*amp*—> Δ*cydAB*. Following knockout of both Δ*cyoABCDE* and Δ*cydAB*, a growth defect in LB-Miller medium was observed as previously reported [31]. Growth of these strains was observed to be substantially more robust on a more enriched medium; hence, BHI was used for the routine propagation of these strains.

**Table 2:** Primers and oligos used for construction of mutant strains

Name	Sequence 5’—> 3’
cyo_up_fwd	agatggagtcagagagcg
cyo_up_rev	tcgttaaatgtaacacaacctctcagttaaaaag
cyo_down_fwd	ggttgtgttacatttaacgacctcaattcc
cyo_down_rev	ctggattatctggcgctac
app_up_fwd	cgttggcccatgaattac
app_up_rev	ctgctccttacatgcgcactcctgtagg
app_down_fwd	agtgcgcatgtaaggagcaggaacaatg
app_down_rev	ttcgtcaacaatccgtcg
cyd_up_fwd	ccaatgaaacaataagtgac
cyd_up_rev	tccttacttacatcatgactccttgctc
cyd_down_fwd	agtcatgatgtaagtaaggagctaaaaatgtg
cyd_down_rev	gaatgagggcaagttaag
ygiN_up_fwd	GACAGCGACTACGATGTC
ygiN_up_rev	aatgtatttaCATGGTTAACTCCTTCTAAAGC
ygiN_down_fwd	gcattggtaaATTCGTATCCTGCAGC
ygiN_down_rev	TGGTATTGGCGCTTCG
ygiN_AmpR_fwd	gttaaccatgTAAATACATTCAAATATCTATCCGC
ygiN_AmpR_rev	ggatacgaatTTACCAATGCTTAATCAGG
cyo_gRNA_A_fwd	tagtGCCTAGCGAATACAACCAGG
cyo_gRNA_A_rev	aaacCCTGGTTGTATTCGCTAGGC
cyo_gRNA_B_fwd	tagtGTTCTGGATGGTGATGACAG
cyo_gRNA_B_rev	aaacCTGTCATCACCATCCAGAAC
cyo_gRNA_C_fwd	tagtAAACGTACATCTGAATAACG
cyo_gRNA_C_rev	aaacCGTTATTCAGATGTACGTTT
app_gRNA_A_fwd	tagtGCAATACCGAAGACTACCGG
app_gRNA_A_rev	aaacCCGGTAGTCTTCGGTATTGC
app_gRNA_B_fwd	tagtACCTACCCACGCTTGCAACG
app_gRNA_B_rev	aaacCGTTGCAAGCGTGGGTAGGT
app_gRNA_C_fwd	tagtAGTACAACCGGTAAAACTGG
app_gRNA_C_rev	aaacCCAGTTTTACCGGTTGTACT
cyd_gRNA_A_fwd	tagtTATCTGCGTCTGTACTACAC
cyd_gRNA_A_rev	aaacGTGTAGTACAGACGCAGATA
cyd_gRNA_B_fwd	tagtTGCAACCAGAATCCACAGTG
cyd_gRNA_B_rev	aaacCACTGTGGATTCTGGTTGCA
cyd_gRNA_C_fwd	tagtTGAGAAGATGAGATCGCCTG
cyd_gRNA_C_rev	aaacCAGGCGATCTCATCTTCTCA

### Cell treatments and immunoblotting

Analysis of HIF-1α by immunoblotting was performed as described previously [19, 32]. Briefly, cells plated in 6-well plates were fed with fresh cell culture medium and treated for the given time period, typically 6 h, with the given strain at a standardized inoculum of 4 × 10^7^ colony forming units (CFU) per well or with 300 μM cobalt chloride (CoCl_2_) as a positive control for HIF-1α stabilization. The PHD inhibitor (HIF stabilizer) IOX-4 (Selleckchem # S6684) was also used in some circumstances at 30 μM to stabilize HIF. In some cases, bacteria were killed by heating at 65 °C or by treating with glutaraldehyde at 5% (w/v) final concentration for approx. 2 h and 15 min each. Confirmation of killing efficacy was confirmed by plating undiluted, treated cells onto LB-Miller agar and incubating overnight at 37 °C—in either case, the absence of colonies was observed indicating sterilization of cultures. Prior to treatment of mammalian cells, glutaraldehyde-treated bacteria were thoroughly washed with PBS to remove residual glutaraldehyde. In other cases, 0.4 μm pore size cell culture inserts (Greiner #657640) were placed above the cells, with 1 mL of medium added to the inner “apical” chamber and with/without the same inoculum of EcN that wells without inserts received. After the given time, plates were placed on ice and medium aspirated. Cells were then immediately lysed using freshly made ice-cold lysis buffer, consisting of 1 × Laemmli sample buffer (Bio-Rad #1610747), 100 mM DTT (Sigma-Aldrich #D0632) freshly constituted in ultra-pure water, 1 × HALT Protease Inhibitor Cocktail (Thermo #78438), and 0.5 mM EDTA. Lysates were transferred to 1.5 mL microcentrifuge tubes and sonicated to reduce viscosity, then frozen at −20 °C until needed for analysis. Lysates were separated by SDS-PAGE using 4–20% gradient pre-cast gels (Bio-Rad #4568094 or 4568096), then transferred to 0.2 μm PVDF using a Bio-Rad Trans Blot Turbo instrument and RTA Mini Kits (Bio-Rad #1704272). Subsequent blots were blocked for one hour at room temp. with 5% (w/v) nonfat dry milk (Bio-Rad #1706404) in tris-buffered saline + 0.1% (v/v) Tween 20 (TBS-T) (“blocking buffer”) and incubated with a primary antibody overnight at 4 °C. Primary antibodies used in this study for immunoblotting were mouse anti-HIF1A (BD #610959, 1/500), rabbit anti-HIF1A (Cell Signaling #36169S, 1/1000), rabbit anti-ALDOC (Cell Signaling #81944S, 1/1000), and rabbit anti-ACTB (Abcam #ab8227, 1/10,000) and were diluted in blocking buffer. Blots were then washed repeatedly with TBS-T and incubated with the appropriate HRP-conjugated secondary antibody (MP Bio. #0855676, 0855550) diluted 1/10,000 in blocking buffer for one hour at room temperature. Blots were then further washed with TBS-T and developed using Clarity ECL reagent (Bio-Rad #1705061), then imaged using a Bio-Rad ChemiDoc MP instrument. Presented results are representative of at least three experimental repetitions. Treatment of cells with *L. lactis* followed the same procedure, except that ~6.67 × 10^5^ CFU was added to cells in the presence or absence of 20 μg/mL hemin as described previously [33]. Densitometry was performed using Fiji, with all data presented after normalization to β-actin and relative to control-treated samples [34].

### In vitro RNA purification, quantitative PCR, and gene expression analysis

Cells were treated similarly as for immunoblotting except that treatment time was extended to 8 h and inoculum was approximately 10^7^ CFU/well. At the end of the treatment time, medium was aspirated and 1 mL of cold TRIzol reagent (Thermo #15596026) was added to each well. Plates were swirled gently to lyse cells, then lysates were transferred to microcentrifuge tubes and frozen at −80 °C. RNA was purified from TRIzol lysates according to manufacturer’s instructions except that 1-bromo-3-chloropropane was used as the phase separation reagent [35]. Purified RNA was then cleaned up using lithium chloride (Sigma-Aldrich #L7026) precipitation according to established protocols (product info. sheet for Thermo #AM9480). cDNA was then synthesized from RNA using iScript Supermix (Bio-Rad #1708840). Gene expression was measured using Power SYBR Green PCR Master Mix (Thermo #4367659) and with a QuantStudio 3 Real-Time PCR System, using the qPCR primers given in Table 3. Removal of residual gDNA by LiCl cleanup was confirmed using no-RT Control Supermix included with iScript Kit. Primer sequences were obtained from Harvard PrimerBank, with PrimerBank ID numbers listed in Table 3 [36]. Gene expression analysis was conducted using LinRegPCR using *ACTB* as a reference gene [37]. Data are presented as fold change relative to untreated controls following *ACTB* normalization. Results are representative of at least two independent experimental repetitions.

**Table 3:** Quantitative PCR (qPCR) primers used in this study

Gene name	PrimerBank ID	Fwd. Seq. 5’—> 3’	Rev. Seq. 5’—> 3’
Human			
*ACTB*	4501885a1	CATGTACGTTGCTATCCAGGC	CTCCTTAATGTCACGCACGAT
*VEGFA*	284172466c1	AGGGCAGAATCATCACGAAGT	AGGGTCTCGATTGGATGGCA
*PGK1*	183603937c1	TGGACGTTAAAGGGAAGCGG	GCTCATAAGGACTACCGACTTGG
*LDHA*	260099724c1	ATGGCAACTCTAAAGGATCAGC	CCAACCCCAACAACTGTAATCT
*BNIP3*	7669480c1	CAGGGCTCCTGGGTAGAACT	CTACTCCGTCCAGACTCATGC
*BNIP3L*	47078259c1	ATGTCGTCCCACCTAGTCGAG	TGAGGATGGTACGTGTTCCAG
*CA9*	169636419c2	TTTGCCAGAGTTGACGAGGC	GCTCATAGGCACTGTTTTCTTCC
*CKB*	356883060c1	GCTGCGACTTCAGAAGCGA	GGCATGAGGTCGTCGATGG
*CLDN2*	283806633c1	GCCTCTGGATGGAATGTGCC	GCTACCGCCACTCTGTCTTTG
Mouse			
*Actb*	6671509a1	GGCTGTATTCCCCTCCATCG	CCAGTTGGTAACAATGCCATGT
*Vegfa*	6678563a1	GCACATAGAGAGAATGAGCTTCC	CTCCGCTCTGAACAAGGCT
*Ca9*	21314850a1	TGCTCCAAGTGTCTGCTCAG	CAGGTGCATCCTCTTCACTGG
*Ecad*	6753374a1	CAGGTCTCCTCATGGCTTTGC	CTTCCGAAAAGAAGGCTGTCC

### Oxygen consumption experiments

Oxygen consumption experiments were performed using Oxodish OD24 plates (PreSens Precision Sensing GmbH), which measure dissolved oxygen level through a precalibrated fluorescent probe. Overnight cultures of bacterial strains were prepared as described above, then inoculated into DMEM/F12 (Thermo #1133032) after normalization based on OD600 and incubated with gentle rocking at 37 °C. Some wells contained only media as a negative control. Bacteria for inoculation were prepared as described before for cell treatments. The experiment was performed at least twice with similar results in each repetition. For Oxodish involving C2BBe1-bacterial co-cultures, cells were plated directly into Oxodish wells and grown until confluent (cells did not grow on Oxodish sensor spots). Then, medium was refreshed and the given bacterial strain added at an inoculum of ~10^6^ CFU/well. Negative control wells for oxygen consumption received medium containing 2 μM antimycin A and 1 μM oligomycin to inhibit cellular respiration.

### Luciferase reporter experiments

HeLa cells were plated on 6-well plates, then co-transfected approximately 1 day later with pGL4.22-PGK1-HRE::dLUC (Addgene #128095, a gift from Chi Van Dang), which expresses destabilized firefly luciferase from a HIF-1α-responsive promoter, and pGL4.75[hRluc/CMV] (Promega #E6931) as a normalization control, which constitutively expresses *Renilla* luciferase from a CMV promoter [38]. Transfections were performed using FuGENE HD (Promega #E2311) according to the manufacturer’s instructions. One day after transfection, cells were treated in fresh growth medium with either 300 μM CoCl_2_ (positive control) or with 2.7 × 10^6^ CFU/well of the indicated strain of *E. coli* Nissle. Some wells were left untreated as negative controls. Cells were incubated with treatments for 8 h at 37 °C, 5% CO_2_, then luciferase activity was measured using a Dual-Glo Luciferase Assay System (Promega #E2920) according to the manufacturer’s protocol. Luciferase measurements were taken using a Biotek Synergy H1 plate reader. Results are representative of at least two independent experimental repetitions.

### Salmonella invasion assays

Invasiveness of wild-type *Salmonella* Typhimurium SL1344 and the otherwise isogenic mutant SL1344 Δ*invA* was measured as described in detail elsewhere [39]. Briefly, HeLa cells were infected with Salmonella Typhimurium at a multiplicity of infection (MOI) of approx. 151 (SL1344 WT) or 217 (SL1344 Δ*invA*) for 10 min., after which cells were washed and given fresh antibiotic-free medium. At approx. 30 min. post-infection, gentamicin (Lonza #17-518Z) was added to cells at 100 μg/mL to kill extracellular bacteria. At one hour post-infection, medium was aspirated from cells, and cells were thoroughly washed with sterile PBS. Cells were then lysed using PBS + 0.2% (w/v) sodium deoxycholate (Sigma-Aldrich #D6750). Lysates were serially diluted using sterile PBS and plated onto LB-Miller agar containing 100 μg/mL streptomycin. Plates were incubated overnight at 30 °C. Colonies were counted the following day, and colony counts were used to determine the number of intracellular bacteria.

### In vivo colonization, immunohistochemistry, immunofluorescence, and gene expression

Wild-type C57BL/6 mice were maintained at the University of Colorado Anschutz Medical Campus in an AAALAC-accredited (#00235) and USDA-inspected (#84-R-0059) facility. All animal procedures were performed under an approved IACUC protocol (#00182). Broad-spectrum antibiotic treatments were performed as described previously [40]. In brief, fifteen mice (3 males, 2 females per experimental group) were gavaged with 100 μL of a sterile-filtered antibiotic solution containing 2 mg/mL ampicillin (Sigma-Aldrich #A0166), 2 mg/mL gentamicin (Sigma-Aldrich #G1914), 2 mg/mL metronidazole (Sigma-Aldrich #M1547), 2 mg/mL neomycin (Sigma-Aldrich #N6386), and 1 mg/mL vancomycin (Sigma-Aldrich #V2002) in ultra-pure water. Control mice received no antibiotics. Gavages were performed twice per day for five consecutive days, after which mice were given a 24-h period to allow a “wash-out” of antibiotics from the gastrointestinal tract. After this, antibiotic-treated mice were subdivided into three groups. One group received a gavage of 100 μL of 10 mg/mL kanamycin once per day for 2 days. Each of the other two groups received a kanamycin gavage followed several hours later by gavage with ~10^8^–10^9^ CFU of *E. coli* Nissle wild-type or “4KO” respiration-deficient mutant in 100 μL sterile PBS. Both strains contained the pEcCas plasmid as a means of conferring kanamycin resistance—this allowed for accurate determination of bacterial density from fecal pellets and for reduction in re-colonizing microbes following broad-spectrum antibiotic wash-out. Presence of this plasmid had no observable effects on bacterial growth. *E. coli* was grown and prepared in PBS as indicated above. After 2 days of *E. coli* gavages, all mice were euthanized by CO_2_ asphyxiation + cervical dislocation. “Scrapings” of cecal intestinal epithelium were taken as previously described and immediately transferred into ice-cold TRIzol reagent for RNA isolation [41]. RNA was isolated and analyzed by qPCR as described above except a hybrid TRIzol/column (NEB # T2040L) method was used for purification according to the manufacturer’s protocol. Mouse-specific qPCR primers were selected using Harvard PrimerBank and are listed in Table 3, with analysis being performed as done previously [36, 41]. Outlying wells were identified using the ROUT method and Grubbs Test (GraphPad Prism) and excluded only if flagged by both methods. Proximal colon tissue was isolated into 10% neutral buffered formalin (Epredia #5701) for histology. Immunohistochemistry (IHC) against HIF-1α was performed by the CVP Histopathology core at the University of Colorado Anschutz Medical Campus with a Roche Discovery platform, using rabbit anti-HIF1A (Abcam #ab51608, 1/1,000) and 3,3′-Diaminobenzidine (DAB) development. Slides were counterstained using hematoxylin. IHC images were captured using an EVOS M5000 imaging system on “RGB illumination” brightfield mode, with identical exposure settings (Light/Exposure/Gain) and objective/zoom level for each sample. Measurement of DAB staining intensity normalized to nuclei was performed using Fiji image analysis software as described elsewhere [34, 42, 43]. Tissue immunofluorescence was performed as done previously with minor alterations [44]. Briefly, FFPE sections were deparaffinized and immersed in Tris-EDTA buffer, pH 9.0 for antigen retrieval using the wet autoclave method described elsewhere [45]. Tissue sections were blocked using TBS + 1% (w/v) bovine serum albumin (BSA) + 10% (v/v) normal goat serum, then probed overnight at 4 °C with anti-Claudin-2 antibody (Thermo #51–6100, 1:100) diluted in TBS/BSA. Slides were washed and stained with goat anti-rabbit secondary (Thermo #A11011, 1:1000) likewise diluted in TBS/BSA for 1 h at room temp. Tissue autofluorescence was quenched using a commercial kit (TrueView, Vector Laboratories #SP-8400–15), then counterstained with DAPI and mounted. Slides were visualized using an EVOS M5000 microscope and photographed using identical exposure settings (exposure time, gain, etc.). Colonization of bacteria was determined by plating fecal pellets obtained just prior to sacrifice—fecal pellets were homogenized in sterile PBS and plated onto either LB-Miller agar (EcN WT) + 100 μg/mL kanamycin or BHI + 100 μg/mL ampicillin and kanamycin (EcN 4KO). In all mice, colonization after 2 days was > 5 × 10^7^ CFU/g fecal content as determined by plating PBS-suspended fecal pellets onto MacConkey agar (BD #212123) containing 100 μg/mL kanamycin (Fig. S3). No growth was observed on these plates from mice receiving antibiotics only, indicating that the observed growth was specific to the introduced *E. coli* strains.

### Data analysis, statistical testing, and image generation

Data were analyzed using GraphPad Prism 10 or Microsoft Excel. Statistical tests used can be found in the figure legend for the appropriate figure. Figure images were prepared using GraphPad Prism, Microsoft PowerPoint, SnapGene, and/or BioRender.

## Results

**Fig. 1:**
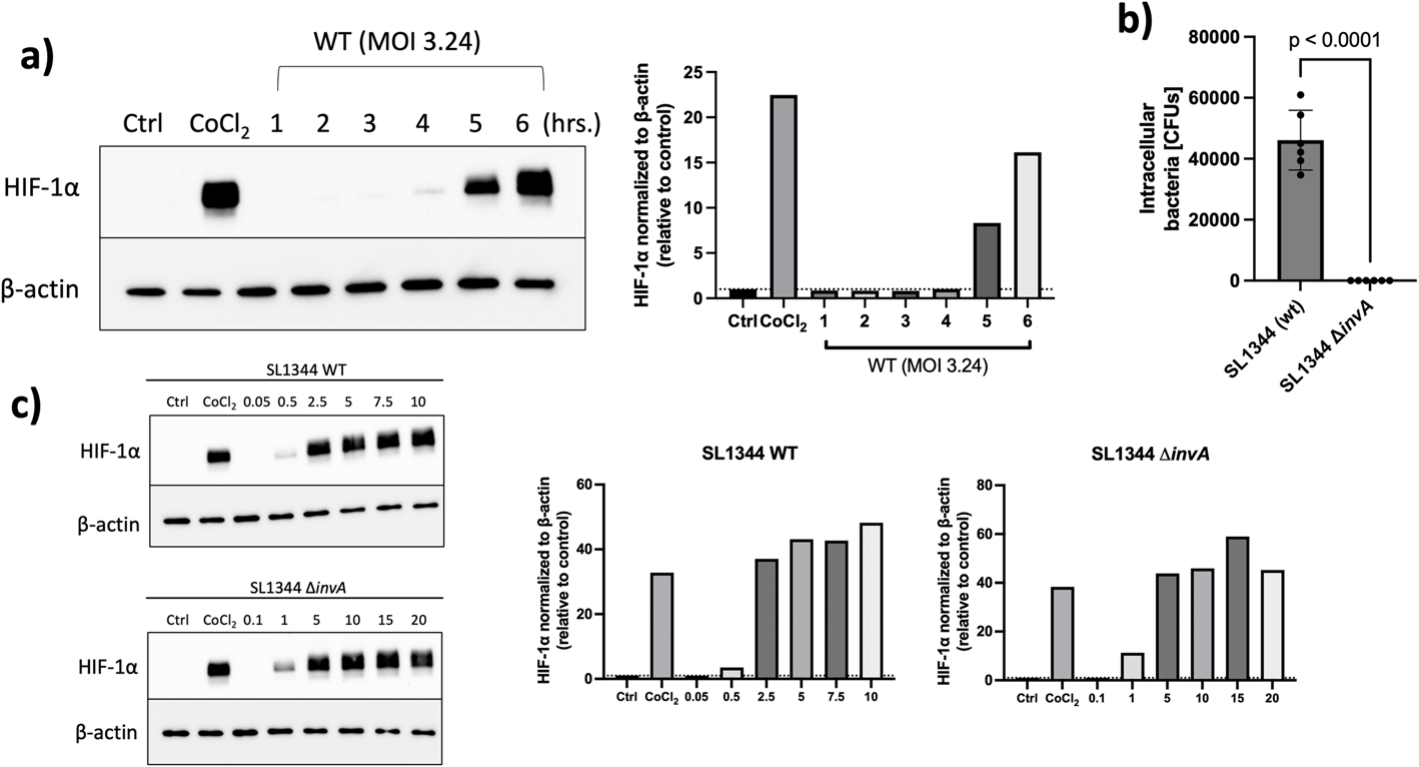
Invasion is not a prerequisite for HIF-1α stabilization by *Salmonella* Typhimurium SL1344. **a** Western blots of HeLa cells treated with wild-type SL1344. “Ctrl” = untreated negative control. “CoCl_2_” = cells treated with 300 μM CoCl_2_ for six hours as a positive control for HIF-1α stabilization. “MOI” = initial multiplicity of infection (bacteria per epithelial cell). **b** Quantification of intracellular bacteria in HeLa cells infected with either wild-type (WT) SL1344 or the invasion-deficient mutant SL1344 Δ*invA*. Significance was calculated using an unpaired t test with Welch’s correction. **c** Western blots of HeLa cells treated at the indicated MOI with either SL1344 WT or SL1344 Δ*invA*. “Ctrl” = untreated negative control. “CoCl_2_” = cells treated with 300 μM CoCl_2_ for six hours. Densitometry analyses are shown to the right of each western blot

Previously, our group sought to characterize the role of HIF-1α in the regulation of anti-bacterial autophagy (“xenophagy”) occurring during infection of epithelial cells by *Salmonella* Typhimurium (STm) strain SL1344 [19]. Similar to this past work, we found that STm SL1344 stabilizes HIF-1α in epithelial cells in a time-dependent manner (Fig. 1a). During previous studies, we found that treatment of intestinal epithelial cells in vitro with STm markedly reduced oxygen tension, a potential mechanism for the observed stabilization of HIF-1α [19]. As STm is a facultative anaerobe and potentially could be respiring oxygen extracellularly, we sought to clarify whether invasion of epithelial cells is a prerequisite for HIF-1α stabilization in a bacterial/epithelial co-culture model. To answer this question, we utilized a mutant strain of STm deficient in InvA, a protein necessary for invasion of epithelial cells (SL1344 Δ*invA*) [46–48]. While we observed efficient invasion of HeLa cells by the wild-type SL1344 strain, we observed essentially no invasion by SL1344 Δ*invA* (Fig. 1b) confirming the invasion defect in this mutant. We then asked whether the SL1344 Δ*invA* strain could stabilize HIF-1α in epithelial cells as its wild-type counterpart does. Treatment of HeLa cells with either wild-type SL1344 or SL1344 Δ*invA* demonstrates stabilization of HIF-1α in a dose-dependent manner and, strikingly, no difference between the invasive and noninvasive strains (Fig. 1c). These results support previous observations that *Salmonella* Typhimurium stabilizes HIF-1α in epithelial cells and suggest that invasion is not a prerequisite for this phenomenon [17, 19].

**Fig. 2:**
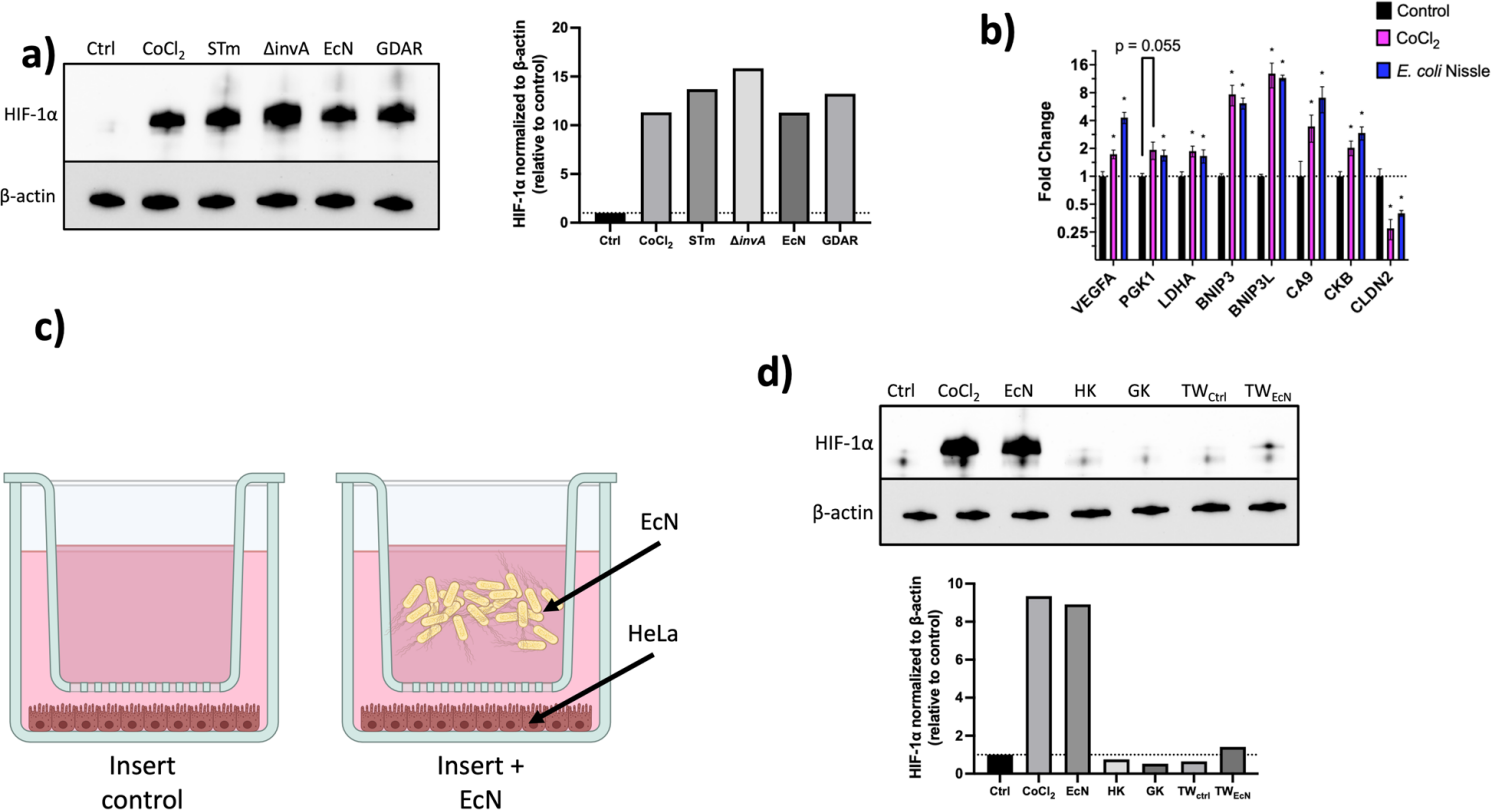
HIF stabilization requires live bacteria in close contact with epithelial cells. **a** Western blots of HeLa cells treated with various enteric bacteria strains. “Ctrl” = untreated negative control. “CoCl_2_” = cells treated with 300 μM CoCl_2_ for six hours. “EcN” = *E. coli* Nissle. “GDAR” = *E. coli* GDAR2-2. **b** Gene expression in C2BBe1 cells treated with either 300 μM CoCl_2_ or *E. coli* Nissle for 8 h. Statistical significance vs. control (untreated) samples was calculated using unpaired t test with Welch’s correction. *p < 0.05. **c** Diagram *E. coli* Nissle–HeLa co-treatment using a cell-culture insert to prevent physical interaction but allowing diffusion of soluble factors. See text for additional details. Made using BioRender. **d** Western blots of HeLa cells treated as indicated for six hours. “HK” = heat-killed EcN. “GK” = glutaraldehyde-killed EcN. “TW_ctrl_” = HeLa cells incubated with a cell-culture insert and no bacteria as a control. “TW_EcN_” = HeLa cells incubated with a cell-culture insert and EcN as depicted in panel **c**. Densitometry analyses shown to the right of each western blot

As STm mutants deficient in cellular invasion have previously shown attenuated pathogenicity in vivo, we then asked whether closely related, nonpathogenic bacteria might also stabilize HIF-1α in vitro [49]. To this end, we examined the ability of *Escherichia coli* to stabilize HIF-1α using commensal strains isolated from either human (*E. coli* Nissle, EcN) or mouse (GDAR2-2) microbiota [23, 50]. We found that both EcN and GDAR2-2 robustly stabilized HIF-1α in HeLa epithelial cells, similar to that of wild-type SL1344 and SL1344 Δ*invA* (Fig. 2a). We then continued our focus on commensal *E. coli* using EcN, as this bacterium has received considerable recent attention as a potential chassis for detection and treatment of human gastrointestinal disease [51–53]. We found that treatment of C2BBe1 epithelial cells with EcN induced numerous classic HIF targets such as *VEGF*, *BNIP3*, and *LDHA* (Fig. 2b), indicating that stabilized HIF was transcriptionally active [54–58]. Importantly, EcN induced expression of the “pro-barrier” gene *CKB* and downregulated the “leaky” claudin gene *CLDN2*, the expression of which inversely correlates with barrier function [21, 59]. We then sought to clarify the mechanism by which EcN might be activating HIF in epithelial cells; specifically, we asked whether living cells were necessary as opposed to killed cells. To this end, we treated HeLa cells with either living EcN, EcN killed by heating at 65 °C, or EcN killed by treatment with glutaraldehyde. We also asked whether EcN might be secreting a soluble factor or producing a metabolite that could activate HIF in the epithelium. To test this hypothesis, we co-incubated living EcN with epithelial cells separated by a 0.4-μm pore-permeable cell-culture insert, which would permit the diffusion of soluble factors but restrict intimate cell-bacterial contact (Fig. 2c). We observed that inactivation of EcN with either heat or glutaraldehyde abolished the stabilization of HIF-1α; likewise, physical separation of EcN from the epithelium prevented an increase in observed HIF-1α (Fig. 2d). These findings indicate that living bacteria, in close contact with the epithelium, are needed to activate HIF.

**Fig. 3:**
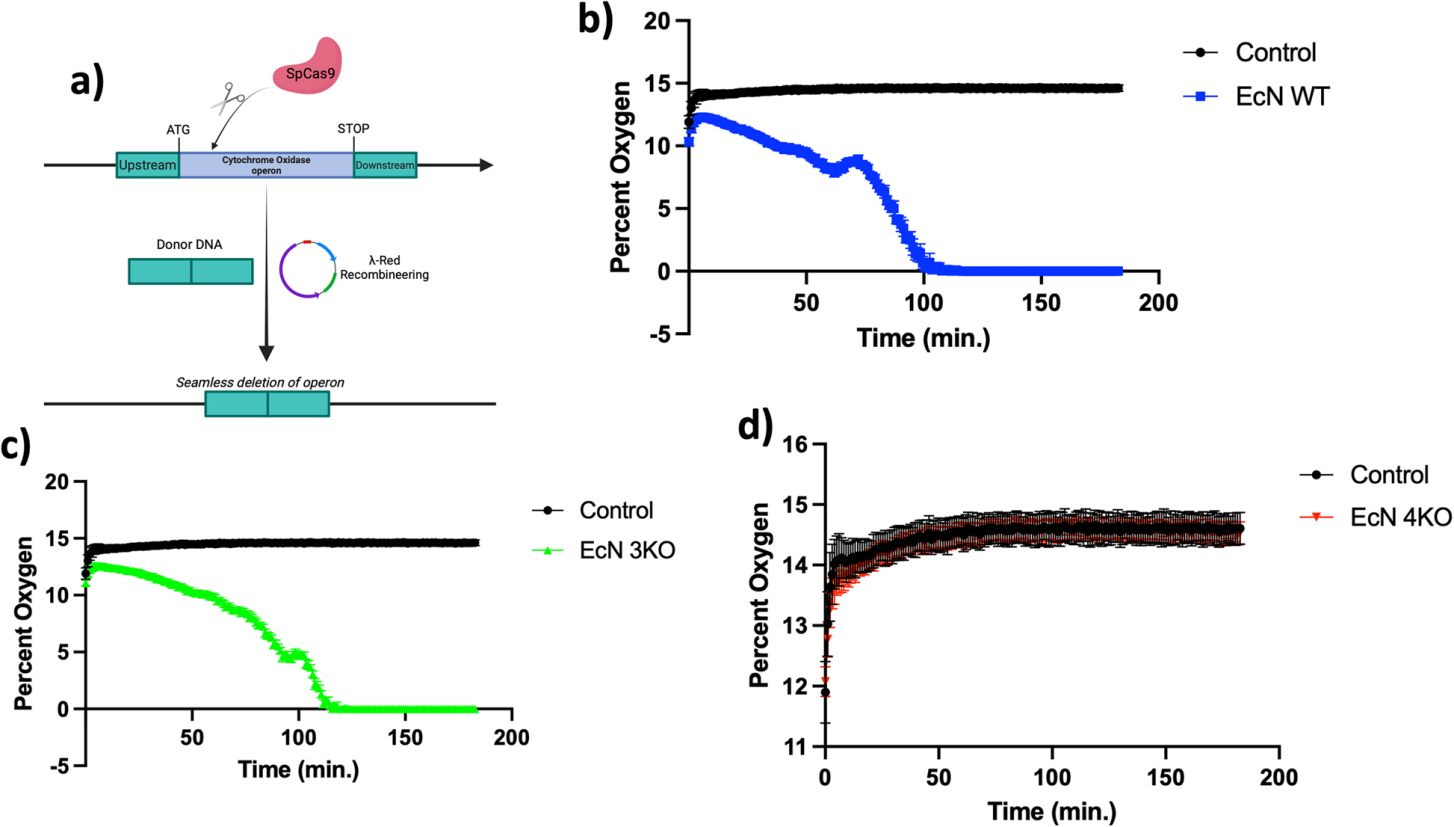
Loss of cytochrome oxidases and YgiN abolishes oxygen consumption in *E. coli* Nissle. **a** Diagram of gene deletion strategy in EcN using CRISPR/Cas9 + λ-Red recombineering. Made using BioRender. **b** Oxodish measurement of extracellular dissolved oxygen in wild-type EcN. **c** Oxodish measurement of extracellular dissolved oxygen in EcN “3KO” (Δ*cyoABCDE* Δ*appCB* Δ*ygiN*::*amp*). **d** Oxodish measurement of extracellular dissolved oxygen in EcN “4KO” (Δ*cyoABCDE* Δ*appCB* Δ*ygiN*::*amp* Δ*cydAB*). In panels **b **through **d**, “control” represents wells containing only DMEM/F-12 and no bacteria. Oxygen consumption is significantly different from controls in wells containing EcN WT and EcN 3KO but not EcN 4KO (two-way ANOVA)

Given our previous findings that live, cell-associated bacteria are required for stabilization of HIF-1α, we then asked what bacterial processes might drive HIF-1α activation in epithelial cells. As HIF-1α is stabilized classically by hypoxia, and as *E. coli* are facultative anaerobes, we hypothesized that HIF-1α stabilization in epithelial cells might be driven by bacterial aerobic respiration. As many of the classic electron-transport-chain inhibitors, such as oligomycin, antimycin A, and rotenone, are ineffective at fully blocking *E. coli* aerobic respiration, we turned to a genetic approach to abolish *E. coli* oxygen consumption [60–62]. Previous work suggests that deletion of *E. coli* cytochrome oxidases (Cyo, Cyd, and App/Cbd/Cyx), the central enzyme complexes that reduce molecular oxygen to water in the bacterial inner membrane, eliminates aerobic respiration and imparts a fermentative growth phenotype [31, 63, 64]. To this end, we generated a strain of EcN which lacks all three cytochrome oxidases (Δ*cyoABCDE* Δ*appCB* Δ*cydAB*) through a combination of λ-Red recombineering + CRISPR/Cas9 negative selection for bacteria that fail to undergo homologous recombination at the appropriate locus (Fig. 3a). In addition to the cytochrome oxidases, the gene *ygiN* was deleted as well: this gene encodes a probable quinol monooxygenase that uses molecular oxygen to oxidize quinols to quinones [65]. Previous reports suggest that *ygiN* is upregulated in the absence of cytochrome oxidases, resulting in a non-negligible level of oxygen consumption in otherwise fermentative cells [31, 64]. We found this to be true as well and deleted *ygiN* through replacement of the wild-type coding DNA sequence with an ampicillin/carbenicillin resistance cassette (Δ*ygiN*::*amp*). Although at least one group has observed absence of oxygen utilization in a YgiN-expressing strain, we found that deletion of *ygiN* was necessary to completely abolish oxygen consumption [63]. The resulting EcN “quadruple” knockout (Δ*cyoABCDE* Δ*appCB* Δ*ygiN*::*amp* Δ*cydAB*) was termed “EcN 4KO” and demonstrated poor growth in LB-Miller medium, as previously found for *E. coli* MG1655 with a similar genotype [31, 64]. We observed good growth in brain-heart infusion (BHI), however, and used this medium for growth of EcN 4KO. We also generated “EcN 3KO” as a control, which was identical to EcN 4KO except that it still expressed the cytochrome bd-I oxidase Cyd (Δ*cyoABCDE* Δ*appCB* Δ*ygiN*::*amp*). All strains that were used at the same time as EcN 4KO were grown in BHI to control for any potential effects of bacterial medium on observed phenotype. We then sought to demonstrate that EcN 4KO was deficient in oxygen respiration. To do so, we incubated EcN wild-type (WT), EcN 3KO, and EcN 4KO in Oxodish plates as done previously to measure extracellular oxygen levels [19]. As shown in Fig. 3b, EcN WT robustly consumes dissolved oxygen, rendering the Oxodish wells hypoxic within two hours. EcN 3KO, which contains a functional cytochrome oxidase (Cyd), similarly consumes oxygen rapidly but with slightly slower kinetics (Fig. 3c)—this could be due to the lower efficiency of the cytochrome bd-I oxidase in generating a proton motive force versus the main aerobic cytochrome bo oxidase [66]. In contrast to EcN WT and EcN 3KO, the “quadruple mutant” EcN 4KO shows no appreciable oxygen consumption and is not significantly different at any data point versus the uninoculated control wells (Fig. 3d, p > 0.05 by two-way ANOVA).

**Fig. 4:**
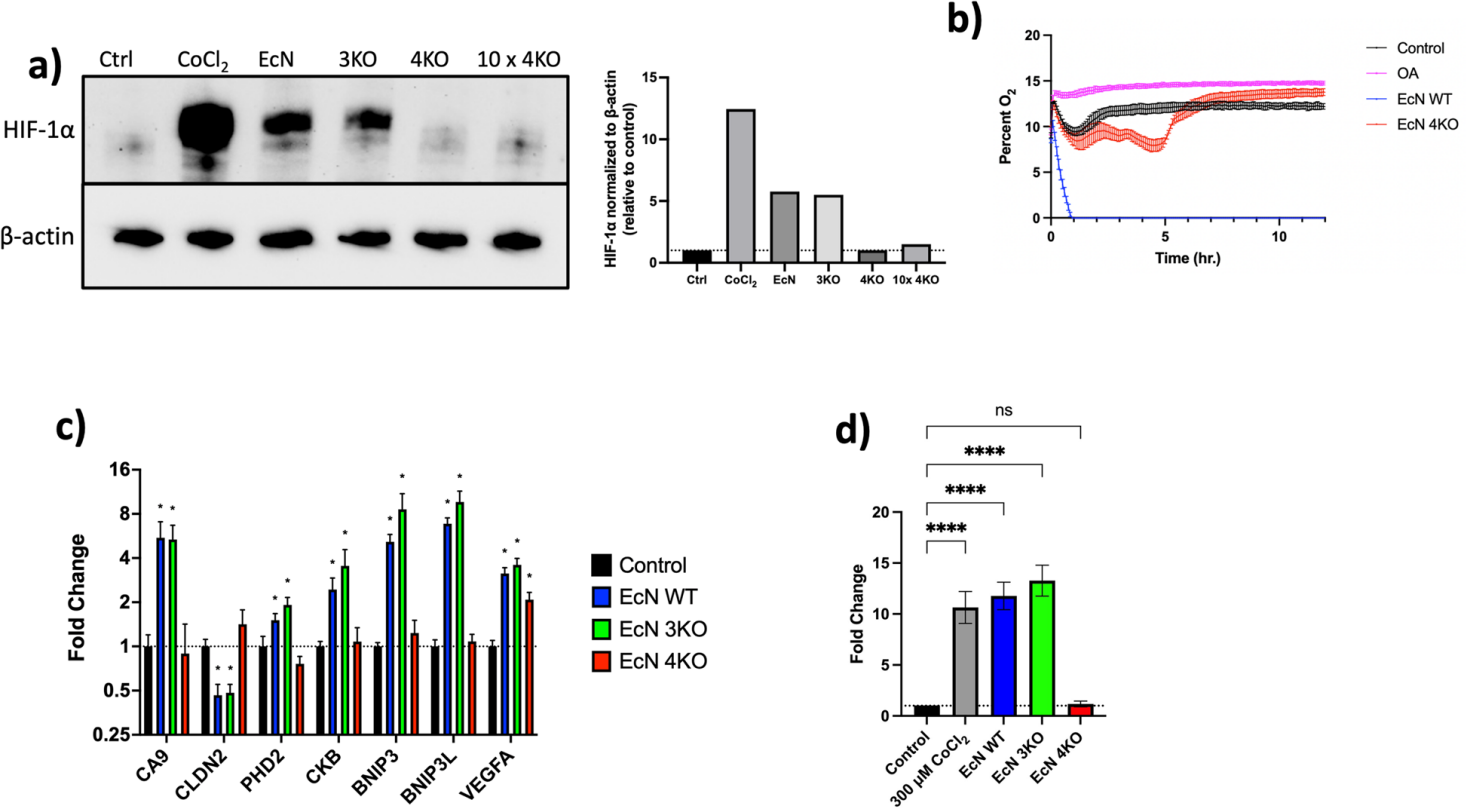
HIF stabilization by *E. coli* Nissle is dependent on oxygen respiration. **a** Western blots of C2BBe1 cells treated with the indicated strain for 6 h. “10 × 4KO” indicates that ten-fold of the standard inoculum of EcN 4KO was applied to these cells (~ 4 × 10^8^ CFU). **b** Oxodish analysis of C2BBe1-EcN co-culture. “OA” = antimycin A + oligomycin (see Materials and Methods). **c** qPCR analysis of gene expression in C2BBe1 cells treated as indicated for 8 h. Statistical significance vs. control (untreated) samples was calculated using unpaired t test with Welch’s correction. *p < 0.05. **d** Luciferase expression in HeLa cells co-transfected with HRE-firefly luciferase and constitutively expressed *Renilla* luciferase (normalization control), followed by treatment with the given strain. Statistics calculated pair-wise vs. untreated control using unpaired t tests with Welch’s correction, with **** =* p* < 0.005 and “ns” = not significant (*p* ≥ 0.05). Densitometry analyses are shown to the right of the western blot in Fig. 5a

**Fig. 5:**
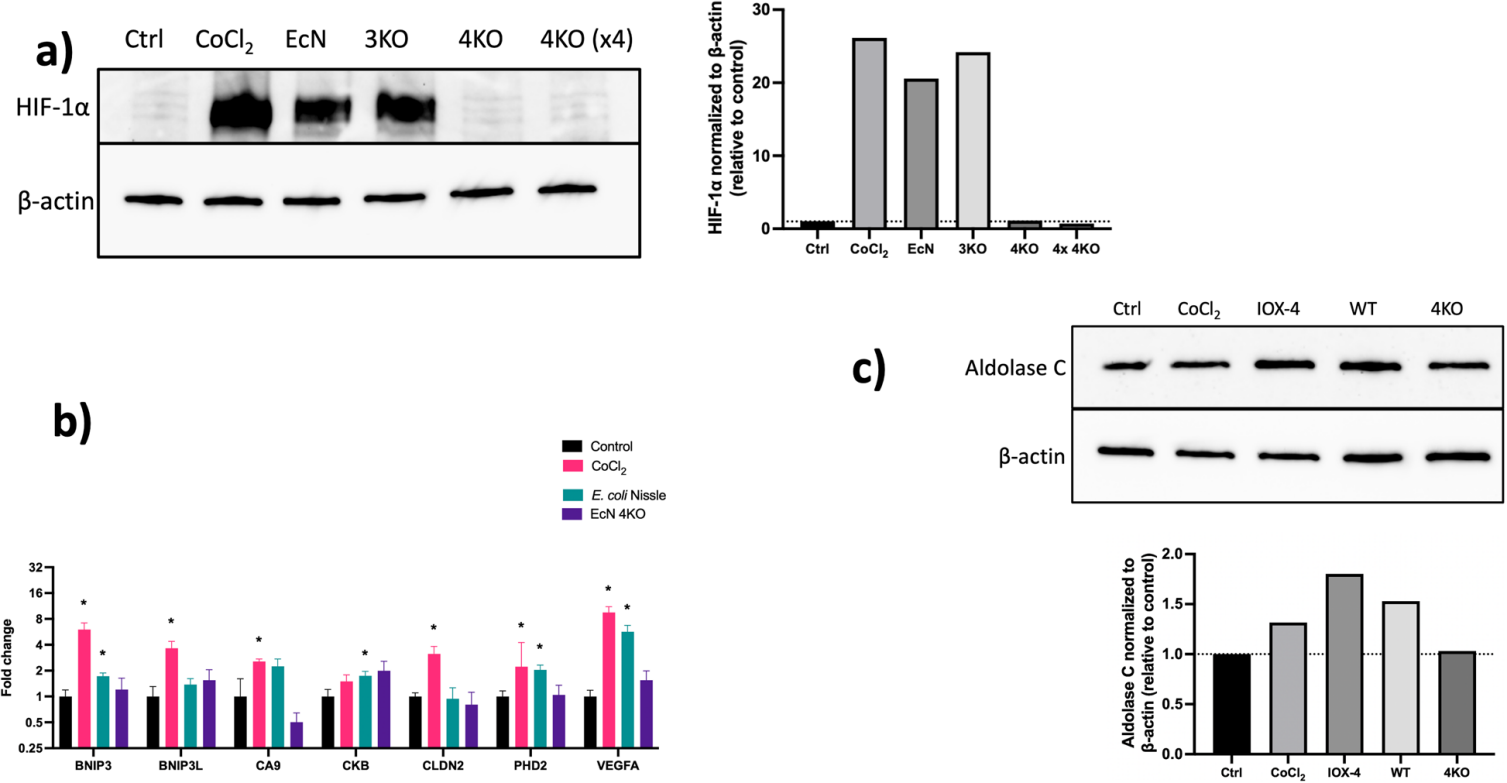
Dependence of HIF stabilization on bacterial oxygen respiration is preserved in normal (non-transformed) intestinal epithelial cells. **a** Western blots of hIEC-6 intestinal epithelial cells treated as indicated for six hours. “4KO × 4” indicates that a four-fold greater inoculum of EcN 4KO was used to treat these cells. **b** qPCR of hIEC-6 cells treated as indicated for 8 h. Statistics calculated pair-wise vs. control using unpaired t tests with Welch’s correction. * = *p* < 0.05 and “ns” = not significant. **c** Western blot analysis of aldolase C expression in hIEC-6 cells treated for 18 h. with the given strain, 300 μM CoCl_2_, or 30 μM IOX-4. Densitometry analyses are shown adjacent to each western blot

**Fig. 6:**
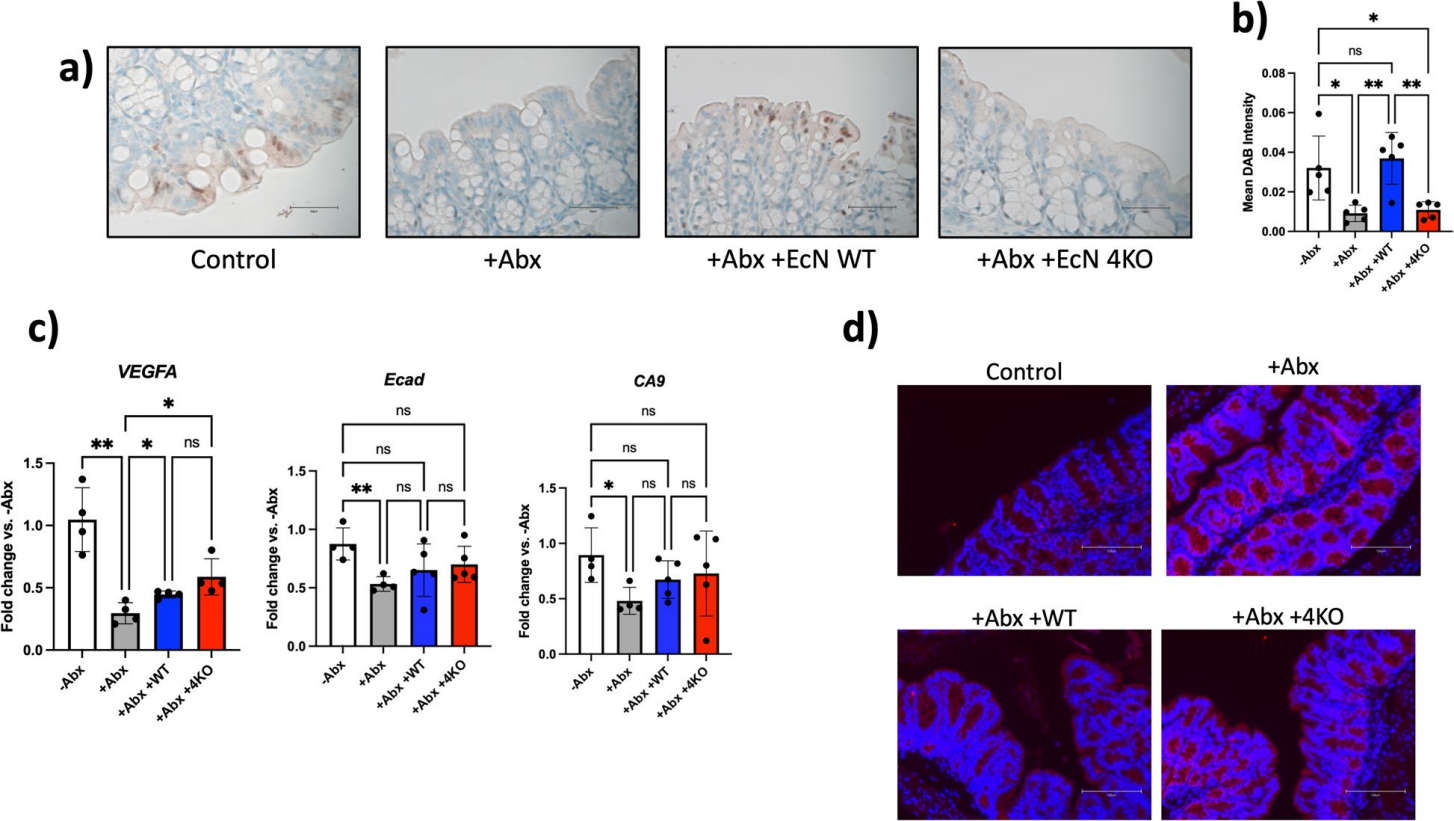
Antibiotic-mediated loss of intestinal epithelial HIF-1α is rescued by EcN WT but not EcN 4KO. **a** Representative IHC images of proximal colon tissue stained for HIF-1α from untreated (control) mice or mice treated with broad-spectrum antibiotics (+ Abx) and with/without EcN WT (+ Abx + EcN) or EcN 4KO (+ Abx + EcN 4KO). **b** Measurement of DAB intensity in IHC slides normalized to nuclei as described in the text. **c** qPCR analysis of gene expression in cecal scraping from the indicated treatment groups. In panels **b** and **c**, statistics were calculated using one-way ANOVA with Welch’s correction for unequal standard deviations. *p < 0.05, **p < 0.01, “ns” = not significant (*p* ≥ 0.05). **d** Immunofluorescent staining for claudin-2 (red) in proximal colon tissue from mice from the given treatment groups. Tissue was counter-stained for DAPI (blue), and slides were photographed using identical exposure conditions. Scale bar = 125 μm

We next assessed the potential for the EcN 4KO mutant to stabilize HIF-1α in intestinal epithelial cells. We found that this mutant was incapable of stabilizing HIF-1α in C2BBe1 cells, unlike both the wild-type EcN and the oxygen-respiring EcN 3KO mutant (Fig. 4a). Use of a tenfold increased inoculum of EcN 4KO did not result in restoration of HIF-1α stabilization, suggesting that the absence of HIF-1α is not due to a growth defect of EcN 4KO but rather due to altered bacterial metabolism (Fig. 4a). HIF stabilization was absent even when a “dose–response” treatment of EcN 4KO was performed at a very high inoculum (> 10^8^ CFU), thus demonstrating that the stabilization of HIF is strongly linked to the respiration phenotype of these bacteria (Fig. S1). We further confirmed that the presence of *E. coli* Nissle WT, but not the 4KO strain, generates a hypoxic microenvironment by co-culturing C2BBe1 cells and the given bacterial stain in an Oxodish plate, followed by measurement of dissolved O_2_ (Fig. 4B). In addition, we used 2 μM antimycin A + 1 μM oligomycin as controls to inhibit endogenous cellular oxygen consumption in the absence of bacteria, thereby demonstrating that the shifts in dissolved oxygen observed are not solely due to respiration by the C2BBe1 cells (Fig. 4b). Additionally, we observed no induction of HIF-1α target genes by qPCR in C2BBe1 cells treated with EcN 4KO as compared with cells treated with EcN WT or EcN 3KO, further supporting the observation that HIF-1α signaling is not induced by treatment with the EcN 4KO mutant (Fig. 4c). Interestingly, we did observe a significant increase in *VEGFA* expression in the EcN 4KO-treated cells versus control, untreated cells, despite no significant increase in other HIF-1α target genes assayed. We hypothesize that the increased expression of *VEGFA* in this treatment group could be due to the production of lactate from the EcN 4KO bacteria: previous reports indicate that deletion of all three cytochrome oxidases from *E. coli* results in substantial lactate production during aerobic fermentation, and it has also been shown that lactate induced VEGFA transcription/secretion in a variety of cell types and tissues [31, 64, 67–69]. It is worth noting, however, that the increase in *VEGFA* expression due to EcN 4KO treatment is significantly lower than that of the EcN WT and EcN 3KO treatments, indicating that HIF-1α has a positive contribution towards *VEGFA* expression in EcN-treated epithelial cells. We also performed luciferase reporter assays using HeLa cells transfected with plasmids expressing either firefly luciferase driven by hypoxia response elements (HREs, derived from the HIF-1α target gene *PGK1* promoter) and *Renilla* luciferase constitutively expressed under a cytomegalovirus (CMV) promoter (normalizing control). Treatment of these co-transfected HeLa cells with 300 μM CoCl_2_, EcN WT, or EcN 3KO led to a significant induction of *Renilla*-normalized firefly luciferase signal relative to untreated, transfected control cells (Fig. 4d), indicating an increase in HRE-dependent transcriptional activity. Yet, treatment of these same cells with EcN 4KO resulted in no significant increase in *Renilla*-normalized firefly luciferase signal (Fig. 4d), further demonstrating that EcN 4KO does not drive HIF-1α-dependent gene expression. We also sought to confirm our findings using non-transformed hIEC-6 intestinal epithelial cells. We found that, similar to our previous observations, EcN stabilizes HIF-1α in hIEC-6 cells in a manner dependent on its ability to respire oxygen (Fig. 5a). An increase in the initial inoculum of EcN “4KO” (EcN 4KO × 4, last lane, Fig. 6a) did not restore HIF-1α stabilization in line with our previous observations (Fig. 4a). Additionally, hIEC-6 cells treated with EcN showed induction of HIF target genes similar to our previous findings (Fig. 5b); this induction was likewise dependent on the ability of EcN to respire oxygen as gene induction was not observed in the EcN 4KO strain. We also observed an induction of the glycolytic enzyme aldolase C in hIEC-6 cells treated with CoCl_2_, the HIF-stabilizing compound IOX-4, or EcN WT but not EcN 4KO (Fig. 5c). As aldolase C has been previously shown to be a highly conserved and reliable HIF target gene [70], we interpret this as further evidence of the activation of the HIF transcriptional pathway in EcN WT-treated cells but not in their 4KO-treated counterparts. Finally, we asked whether our observations regarding HIF were limited to *E. coli* and gram-negative bacteria. To answer these questions, we utilized *Lactococcus lactis* MG1363, a heme auxotroph that nevertheless possesses an intact electron transport chain and a functional, heme-dependent cytochrome oxidase. When exogenous heme is supplemented to this organism, it is able to respire oxygen; elsewise, it behaves as an aerotolerant anaerobe [33]. We found that a low inoculum (~ 6.67 × 10^5^ CFU) of *L. lactis* induces HIF stabilization in HeLa cells but only when hemin is added to the culture medium, in agreement with past studies (Fig. S2). Hemin itself had no effect on HIF stabilization at the concentration used (20 μg/mL). Thus, our observations extend beyond *E. coli* to other organisms capable of respiring oxygen. These results, taken together, illustrate that aerobic respiration in *E. coli* Nissle drives HIF-1α stabilization and transcriptional activity in intestinal epithelial cells, including upregulation of pro-barrier gene targets, and that abolishing oxygen consumption in EcN is sufficient to nullify these observed phenotypes.

Finally, we sought to confirm our findings using an in vivo model of host-microbe interactions. Previous work by our group indicates that intestinal hypoxia is dependent on the gut microbiota, as depleting intestinal bacteria using broad-spectrum antibiotics abolishes tissue hypoxia [40]. Further, treatment of mice with broad-spectrum antibiotics reduces colonic HIF-1α expression and decreases HIF target gene expression [41]. We surmised that HIF stabilization and target gene expression could be partially rescued in antibiotic-treated mice through application of respiring EcN but not the fermentative mutant EcN 4KO. To test this, wild-type mice were treated for 5 days with a broad-spectrum antibiotic cocktail previously shown to dramatically deplete the gut microbiota [40]. Following a 24 h “wash-out” period, mice were then administered kanamycin, kanamycin + EcN WT, or kanamycin + EcN 4KO. The EcN strains used in these studies contained a plasmid encoding for kanamycin resistance, to permit antibiotic selection during colonization. Following 2 days of bacterial gavages and colonization, all mice were euthanized and tissues were processed as indicated in the “Materials and Methods” section. We observed a significant decrease in proximal colonic HIF-1α by immunohistochemistry following antibiotic treatment, which was rescued by colonization with EcN WT but not EcN 4KO (Fig. 6a, b). The extents of colonization were measured by plating serial dilutions of fecal pellet suspensions onto MacConkey agar with kanamycin, with the resulting colony counts showing that both strains readily colonized antibiotic-treated mice (Fig. S3). Further, quantification of gene expression from cecal scrapings indicated that antibiotic treatment reduced expression of HIF target (*Vegfa*, *Ca9*) and pro-barrier (*Ecad/Cdh1*) genes (Fig. 6c). Gene expression was rescued by treatment with EcN WT, indicating activation of HIF signaling. Surprisingly, we also observed increased gene expression in the EcN 4KO-treated groups, including as observed before for *VEGFA* (Fig. 4c), suggesting that alternative pathways separate from HIF may be activated in these mice. Finally, we observed an increase in proximal colon tissue claudin-2 immunostaining in antibiotic-treated mice and EcN 4KO-treated mice, but not in controls and those treated with EcN WT (Fig. 6d). Upregulation of claudin-2 in the intestinal crypts, a phenomenon we observed in our antibiotic-only and EcN 4KO groups, has been previously associated with intestinal inflammation and, indeed, claudin-2 itself has been used as a biomarker for in vivo barrier function and wound healing responses [71, 72]. Taken together, these results implicate bacterial respiration at the luminal surface as a mechanism to drive transcriptionally active HIF stabilization in colonic epithelia.

## Discussion

**Fig. 7:**
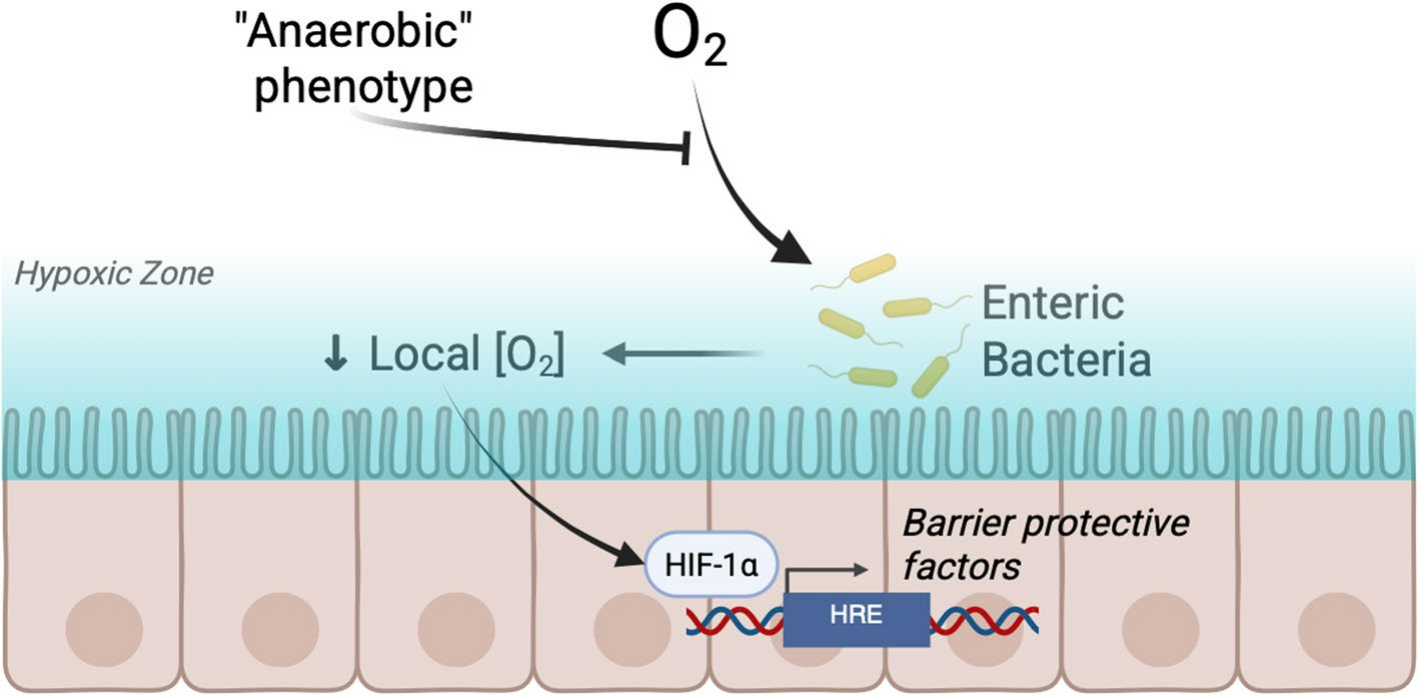
Summary slide of the proposed mechanism by which enteric bacteria stabilize intestinal epithelial HIF in an oxygen-dependent manner. Prepared using BioRender

Although the mechanisms of intestinal hypoxia have been described for some time, namely β-oxidation of short-chain fatty acids and countercurrent vasculature organization, the full contribution of the microbiota to tissue hypoxia (and, therefore, metabolism and gene expression) is not fully understood [73]. Here, we demonstrate a role for commensal, nonpathogenic *E. coli* in the regulation of intestinal homeostasis through activation of HIF-1α. We show that this regulation occurs through host-microbe interactions involving bacterial aerobic respiration, and genetic elimination of oxygen utilization abolishes the observed HIF-1α stabilization and transcriptional activity in epithelial cells. We demonstrate these phenomena in both cancer cell lines and non-transformed intestinal epithelial cells. Finally, we recapitulate our *in vitr*o findings to demonstrate that tissue HIF-1α can be regulated in vivo by *E. coli* Nissle in a manner dependent on its ability to respire oxygen. The systems utilized do have limitations; for example, the in vitro approaches are reductionist and exclude the confounding factors of other gut microbes and/or their metabolites. The specific responses between various differentiated intestinal epithelial cell types—enterocytes, goblet cells, and so forth—versus undifferentiated “stem-like” cells were not specifically modeled by our approaches. We also did not explicitly define the cellular response to bacteria-mediated hypoxia in fully differentiated/confluent, non-proliferative cells due to the previously characterized phenomenon of normoxic HIF stabilization by high cell density [74]. In addition, our in vivo system models these epithelial-bacterial interactions for a short duration in mice that had been raised in an SPF environment prior to antibiotic administration. Nevertheless, our findings are highly consistent and reproducible across multiple cell lines and models, suggesting a conserved underlying mechanism in which epithelial HIF is regulated through bacterial respiration of oxygen. Our current experimental model based on these findings is summarized in Fig. 7.

One interesting observation in our studies is that the bacteria-stabilized HIF-1α is transcriptionally active (e.g., Fig. 2b). This phenomenon was noteworthy as HIF-1α is subject to direct post-translational regulation through modifications such as asparagine hydroxylation by FIH and indirect regulation such as cullin neddylation, both of which regulate HIF-1α’s ability to influence cellular gene expression [75, 76]. The full landscape of HIF post-translational modification is still unclear and is an active field of investigation, yet studies have shown that many of these modifications operate independently of the canonical PHD-FIH axis [77]. For example, during hypoxia HIF-1α becomes SUMOylated resulting in a PHD-independent targeting to VHL [78]. Further, the activity of HIF has been shown to be “fine-tuned” by the differing activities of its regulatory enzymes such as PHDs and FIH, resulting in activation of different cohorts of HIF target genes [79]. The unique nature of bacteria-induced hypoxia hints that a specific HIF transcriptional program might be exerted by the presence of respiring bacteria; future studies should look to identify key HIF target genes regulated through bacterial oxygen consumption.

Previous work regarding host-microbe interactions in the gut has demonstrated that the commensal gut microbiota plays an essential role in excluding opportunistically harmful microbes (pathobionts) as well as professionally pathogenic microorganisms (pathogens) from establishing themselves in the gut microenvironment. This is accomplished through diverse mechanisms, such as competition for scarce nutrients, secretion of interkingdom signaling metabolites, and regulation of host immunological programs [80]. The loss of “beneficial” microbial species and the proliferation of pathobionts and pathogens, often observed during intestinal inflammation and referred to as “dysbiosis”, are accompanied by numerous genetic and environmental alterations in the gut microenvironment [81]. One major dysbiotic alteration observed during these circumstances is the availability of terminal electron acceptors such as molecular oxygen and nitrate, which promote the growth of facultative anaerobes such as those of the *Enterobacteriaceae* family [82]. This diffusion of oxygen into the intestinal lumen occurs in large part due to a weakening of intestinal barrier function and the loss of bacteria-derived SCFAs, which fuel intestinal epithelial metabolism through β-oxidation, thereby reducing luminal oxygen diffusion [83]. Importantly, these SCFAs (especially butyrate) are a major source of intestinal epithelial hypoxia in vivo through their cellular catabolism [40]. Butyrate in particular has been shown to additionally act as a bona fide PHD inhibitor, independently of and in addition to its role as a β-oxidation substrate [41]. The loss of SCFA producers, as can be seen in germ-free mice, results in a striking oxygenation of the intestinal lumen and a loss of HIF activity [40]. These SCFAs are also the principal energy source of the differentiated intestinal epithelium, and their loss results in an energy deficiency that further compromises barrier function, whose maintenance is a highly energy-intensive process [84]. Although restoration of tissue hypoxia promotes barrier-protective signaling programs on its own, restoration of true mucosal homeostasis is dependent on the return of these key microbial-derived metabolites [85].

Interestingly, other groups have observed that oxygen respiration may underlie host-microbe interactions at the intestinal epithelium. Recent work by Litvak et al. demonstrated that competition for oxygen underlies the pathogenesis of *Salmonella enterica* serovar Enteritidis in neonate chicks, and that germ-free animals are protected from infection when colonized with *E. coli* Nissle [86]. This protection was strictly dependent on the ability of EcN to respire oxygen in microaerophilic conditions, as genetic deletion of *cydA* and *appC* resulted in a loss of this protective phenotype. This is in agreement with our findings and those of others, namely that loss of cytochrome oxidases imposes an obligate fermentative phenotype upon *E. coli* [31, 64]. Similarly, blooms of *Candida albicans*, often observed following courses of antibiotics, are dependent on an oxygenated intestinal lumen—the presence of respiring bacteria can combat these fungal expansions in a similar manner as *Salmonella* Enteritidis [87]. Perhaps most intriguing is the reported ability of *E. coli* to scavenge and detoxify host reactive oxygen species (ROS) encountered during intestinal inflammation [88]. Although host-derived nitrates have long been known to be an electron acceptor for various enteric bacteria such as *Salmonella* Typhimurium, recent work by Chanin et al. suggests that the mechanisms for ROS recovery may be widespread amongst enteric bacteria, possibly explaining the well-characterized bloom of Proteobacteria observed during intestinal inflammation [88–90]. Our observations that bacteria-mediated HIF stabilization is not limited to *E. coli*, or indeed even gram-negative bacteria, corroborate these past findings and raise the intriguing question of the wider role of bacterial respiration in the regulation of intestinal epithelial function. Indeed, the healthy microbiota of the average person contains a minor but non-negligible fraction of respiration-capable bacteria such as *Proteobacteria* [91]. It has also been hypothesized that oxygen-respiring strains are the key founding members of the human gut microbiota during infancy, establishing the hypoxic environment that allows for their supersession as the majority by obligate anaerobes [92]. The precise role of *E. coli* and other facultative anaerobes in the healthy adult intestinal tract is not fully understood, but our evidence points towards a function as a secondary sink for luminal oxygen in addition to the intestinal epithelium’s well-documented role.

Previous work by our group and others has demonstrated that HIF-1α plays an essential role in maintaining intestinal homeostasis. Conditional deletion of this protein in intestinal epithelial cells increased the severity of TNBS(2,4,6-Trinitrobenzenesulfonic acid)-induced colitis in a murine model of IBD; likewise, overexpression of HIF-1α through conditional deletion of *Vhl* or treatment with PHD inhibitors likewise improves endpoints in animal colitis models [14–16]. HIF stabilization also occurs as the result of neutrophil transmigration during intestinal inflammation: the movement of neutrophils into the intestinal lumen, as well as the accompanying respiratory burst, is a highly oxygen-dependent process and renders the surrounding mucosa hypoxic [93]. Targeting HIF as a route for treatment of intestinal inflammation has long been speculated, though currently no FDA-approved therapeutics for IBD are available that act through this mechanism [94]. The ability of commensal, noninvasive *E. coli* Nissle to stabilize HIF-1α and potentiate HIF target gene expression suggests that engineering these bacteria to actively consume luminal oxygen may be a viable strategy for future biotherapeutic development. Oral *E. coli* Nissle preparations, including those utilizing engineered strains, have undergone multiple clinical trials in which the treatments were determined to be safe and free from major adverse events [53, 95, 96]. *E. coli* Nissle is genetically tractable, as demonstrated by our work and others, and it is feasible that a strain could be developed that conditionally increases oxygen respiration in the intestinal tract. Such an inducible expression system might be tied to the acidic microenvironment of the inflamed intestine or to the loss of free zinc due to the action of the inflammation-associated protein calmodulin; indeed, expression systems for *E. coli* have already been developed that utilize these environmental signals to regulate gene expression [97, 98]. A strain of *E. coli* Nissle that inducibly “sponges up” luminal oxygen might thus have the multiform effects of inducing HIF signaling (thus, improving intestinal barrier and wound healing), suppressing outgrowth of dysbiotic facultative anaerobes, and permitting re-establishment of SCFA-producing obligate anaerobes. The interaction between such engineered probiotics and the innate immune system, which is activated during the acute inflammatory flares observed in IBD, remains however an unresolved question that will need to be addressed in the development of these next-generation therapeutics [99].

## Conclusions

In summary, our work identifies a novel host-microbe crosstalk pathway whereby commensal bacteria regulate fundamental functions in the intestinal epithelium through oxygen-respiration-dependent HIF activity. These findings indicate that facultative anaerobe members of the gut microbiota may impact intestinal epithelial physiology through hitherto unappreciated mechanisms and suggest that these pathways might be leveraged in the development of novel therapeutic microbes.

## Supplementary Information

Supplementary Material 1: Figure S1. Dose-response treatment of HeLa cells with *E. coli* Nissle 4KO. HeLa cells were treated with an escalating “dose response” of EcN 4KO as described elsewhere in the text, with 300 μM CoCl_2_, or left untreated ("ctrl") for six hours, then whole-cell lysates were prepared for western blot. Densitometry analysis is shown adjacent to the western blot.

Supplementary Material 2: Figure S2. Treatment of HeLa cells with *L. lactis* MG1363 and/or hemin. HeLa cells were treated with ~ 6.67 × 105 CFU of *L. lactis* MG1363 (“LL”) as described in the text and/or 20 μg/mL hemin (“H”) as indicated. Negative-control cells (“ctrl”) were left untreated and positive controls were treated with 300 μM CoCl_2_. Cells were treated for six hours, then whole-cell lysates were prepared for western blot. Densitometry analysis is shown adjacent to the western blot.

Supplementary Material 3: Figure S3. Quantification of *E. coli* Nissle strains in fecal pellets from gavaged mice. Fecal pellets from mice gavaged for two days with the given bacterial strain were fully suspended in PBS by vortexing, then serial dilutions were plated onto MacConkey agar containing 100 μg/mL kanamycin and incubated overnight at 37 °C. Resulting colonies were measured and densities were back-calculated to fecal mass.

## Data Availability

All data generated or analyzed during this study are included in this published article (and its supplementary information files).

## Abbreviations

3KO: *E**. coli* Nissle Δ*cyoABCDE* Δ*appCB* Δ*ygiN*::*amp*
4KO: *E**. coli* Nissle Δ*cyoABCDE* Δ*appCB* Δ*ygiN*::*amp* Δ*cydAB*
Abx: Antibiotics
EcN: *E. coli* Nissle
HIF: Hypoxia-inducible factor
HIF-1α: Hypoxia-inducible factor 1α, *HIF1A*
IBD: Inflammatory bowel disease
PHD: Prolyl hydroxylase
SCFA: Short-chain fatty acid(s)
TNBS: 2,4,6-Trinitrobenzenesulfonic acid
STm: *Salmonella enterica* subsp. *enterica* serovar Typhimurium
WT: Wild-type
