# Supplementary figures and images for: Metabolic host-microbe crosstalk in stabilization of epithelial HIF
Source: Microbiome. 2026 May 30;14:191. doi: 10.1186/s40168-026-02431-8 (PMC13425931; doi:10.1186/s40168-026-02431-8)

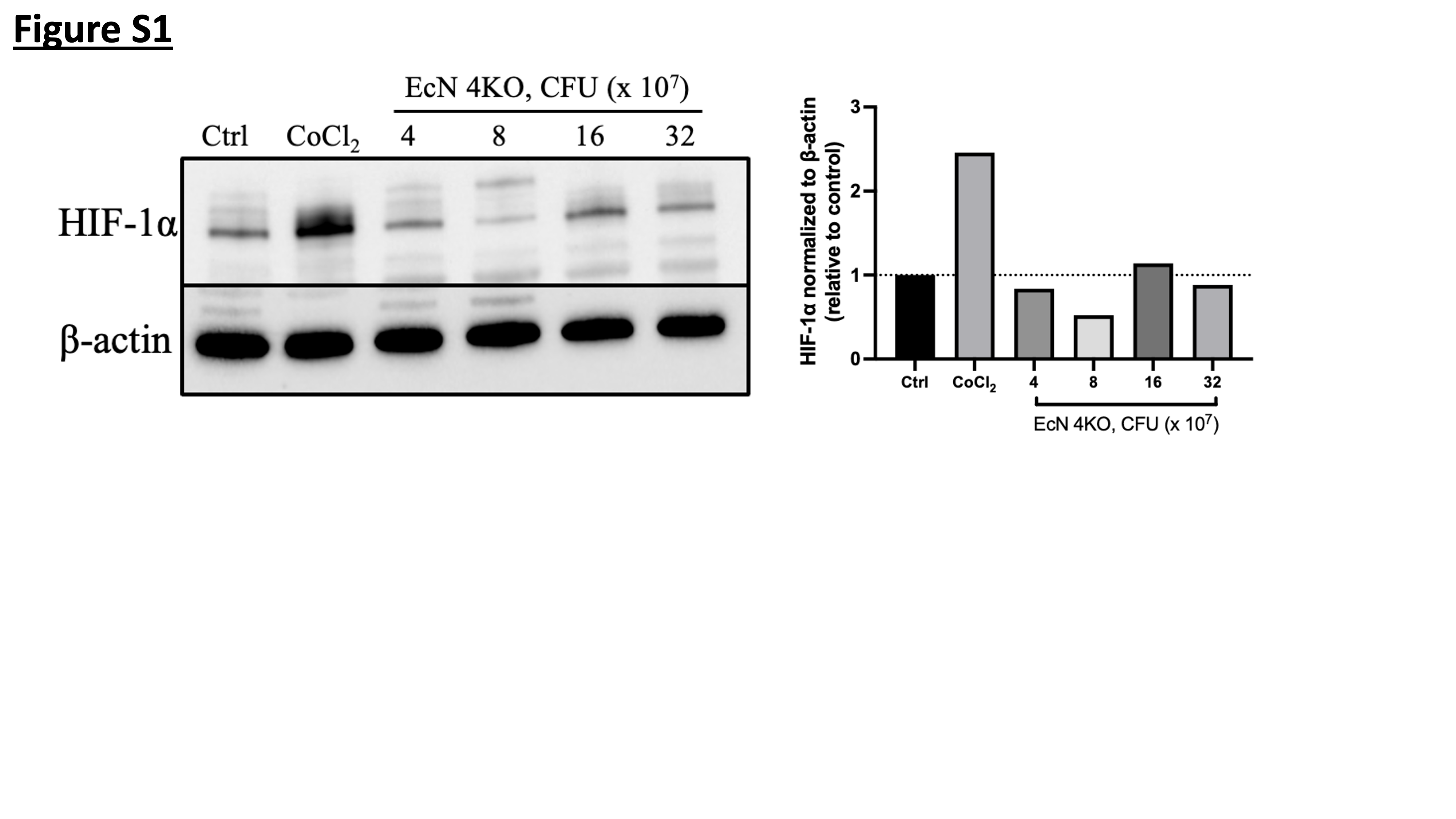

Supplement: Supplementary file 2 — Supplementary Material 1: Figure S1. Dose-response treatment of HeLa cells with E. coli Nissle 4KO. HeLa cells were treated with an escalating “dose response” of EcN 4KO as described elsewhere in the text, with 300 μM CoCl2, or left untreated ("ctrl") for six hours, then whole-cell lysates were prepared for western blot. Densitometry analysis is shown adjacent to the western blot. [file 40168_2026_2431_MOESM1_ESM.png]

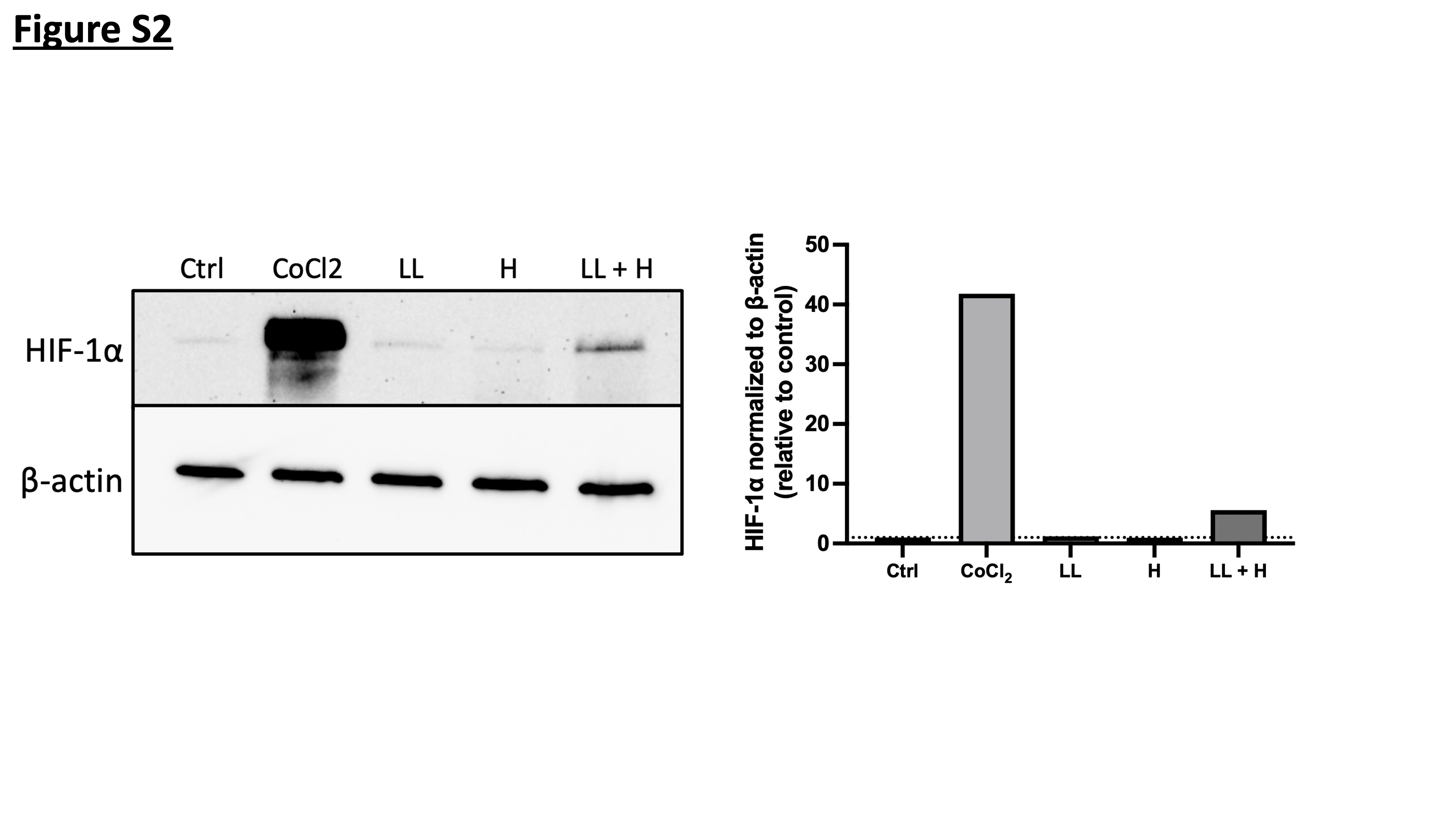

Supplement: Supplementary file 3 — Supplementary Material 2: Figure S2. Treatment of HeLa cells with L. lactis MG1363 and/or hemin. HeLa cells were treated with ~ 6.67 × 10^5^ CFU of L. lactis MG1363 (“LL”) as described in the text and/or 20 μg/mL hemin (“H”) as indicated. Negative-control cells (“ctrl”) were left untreated and positive controls were treated with 300 μM CoCl2. Cells were treated for six hours, then whole-cell lysates were prepared for western blot. Densitometry analysis is shown adjacent to the western blot. [file 40168_2026_2431_MOESM2_ESM.png]

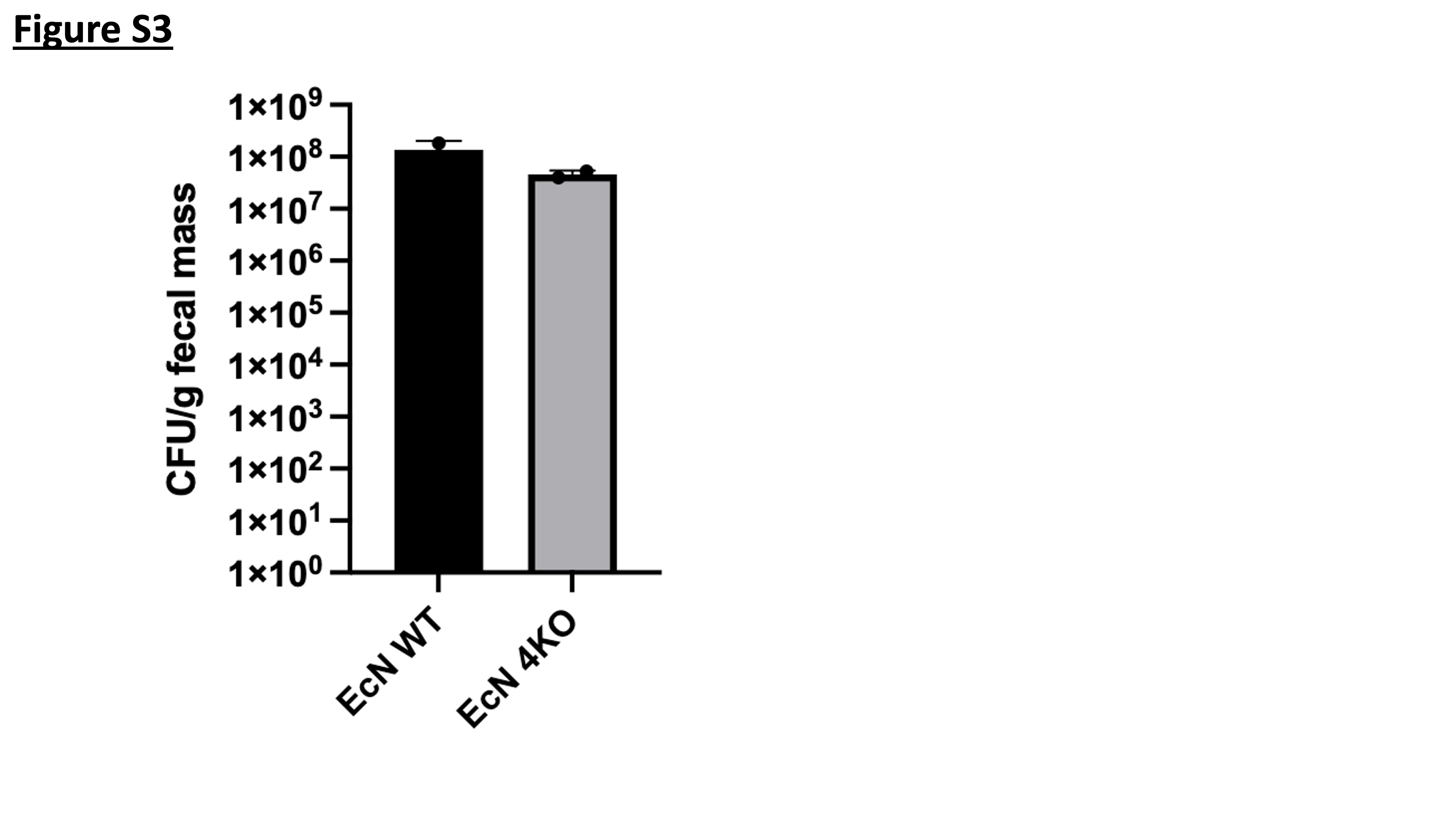

Supplement: Supplementary file 4 — Supplementary Material 3: Figure S3. Quantification of E. coli Nissle strains in fecal pellets from gavaged mice. Fecal pellets from mice gavaged for two days with the given bacterial strain were fully suspended in PBS by vortexing, then serial dilutions were plated onto MacConkey agar containing 100 μg/mL kanamycin and incubated overnight at 37 °C. Resulting colonies were measured and densities were back-calculated to fecal mass. [file 40168_2026_2431_MOESM3_ESM.png]
